# Internet Use and Cognitive Functioning in Later Life: Focus on Asymmetric Effects and Contextual Factors

**DOI:** 10.1093/geroni/igab046.154

**Published:** 2021-12-17

**Authors:** Yijung Kim, Sae Hwang Han

**Affiliations:** The University of Texas at Austin, Austin, Texas, United States

## Abstract

Despite the emerging literature linking information communicative technology (ICT) use and cognitive functioning in later life, whether the association varies as a function of social environment and birth cohort remain an open question. Using nine waves of panel data from the U.S. Health and Retirement Study (2002-2018), we examined within-person asymmetric effects of transitioning into and out of Internet use on cognitive functioning, and whether the associations vary depending on living arrangement and across birth cohorts. Results from the multilevel models indicated that transitioning into Internet use was associated with improved cognitive functioning at a given wave and decelerated cognitive decline over time. Similarly, ceasing to use the Internet was associated with worse cognitive functioning and accelerated cognitive decline. Further, such linkages between Internet use and cognitive functioning were moderated by living arrangement and birth cohort. The detrimental effect of ceasing Internet use was worse for those older adults who live alone. Transitioning into and out of Internet use was unrelated to changes in cognitive functioning among recent HRS cohorts, namely, the War Babies (b:1942-47) and Early Baby Boomers (b:1948-53). These findings highlight the interplay between technology, social environment, and cognitive functioning in later life. The salubrious effects of adopting an ICT technology, such as the Internet, as well as deleterious effects of ceasing to use such technology, underscores the importance of promoting digital literacy and access to ICT technologies among the older adult population.

